# Supplementary material for: Transcriptome Analysis Reveals Vimentin-Induced Disruption of Cell–Cell Associations Augments Breast Cancer Cell Migration
Source: Cells. 2022 Dec 13;11(24):4035. doi: 10.3390/cells11244035 (PMC9776984; doi:10.3390/cells11244035)
Supplement: Supplementary file 1 [file cells-11-04035-s001.zip › DEGs and enrichment analysis.zip!/DEGs and enrichment analysis/WT_VIMvsVector KEGG/ALL/WT_VIMvsVector_ALL.pathway_map.zip!/WT_VIMvsVector_ALL.pathway_map/src/hsa04215.html]

hsa04215


- hsa:332

- Up regulated genes

ENSG00000089685(1.81425481885642)
- hsa:330

- Down regulated genes

ENSG00000023445(-2.12138419902495)

- hsa:596

- Up regulated genes

ENSG00000171791(2.03324203294485)

- hsa:332

- Up regulated genes

ENSG00000089685(1.81425481885642)

- hsa:332

- Up regulated genes

ENSG00000089685(1.81425481885642)

Close
